# Detecting early signs in Duchenne muscular dystrophy: comprehensive review and diagnostic implications

**DOI:** 10.3389/fped.2023.1276144

**Published:** 2023-11-10

**Authors:** Eugenio Mercuri, Marika Pane, Gianpaolo Cicala, Claudia Brogna, Emma Ciafaloni

**Affiliations:** ^1^ Pediatric Neurology, Università Cattolica del Sacro Cuore, Rome, Italy; ^2^ Centro Clinico Nemo, Fondazione Policlinico Universitario Agostino Gemelli IRCCS, Rome, Italy; ^3^ Department of Neurology, University of Rochester, Rochester, NY, United States

**Keywords:** Duchenne muscular dystrophy, delayed diagnosis, time to diagnosis, physical milestones, developmental milestones

## Abstract

Despite the early onset of clinical signs suggestive of Duchenne muscular dystrophy (DMD), a diagnosis is often not made until four years of age or older, with a diagnostic delay of up to two years from the appearance of the first symptoms. As disease-modifying therapies for DMD become available that are ideally started early before irreversible muscle damage occurs, the importance of avoiding diagnostic delay increases. Shortening the time to a definite diagnosis in DMD allows timely genetic counseling and assessment of carrier status, initiation of multidisciplinary standard care, timely initiation of appropriate treatments, and precise genetic mutation characterization to assess suitability for access to drugs targeted at specific mutations while reducing the emotional and psychological family burden of the disease. This comprehensive literature review describes the early signs of impairment in DMD and highlights the bottlenecks related to the different diagnostic steps. In summary, the evidence suggests that the best mitigation strategy for improving the age at diagnosis is to increase awareness of the early symptoms of DMD and encourage early clinical screening with an inexpensive and sensitive serum creatine kinase test in all boys who present signs of developmental delay and specific motor test abnormality at routine pediatrician visits.

## 1. Introduction

Duchenne muscular dystrophy (DMD) is the most common form of inherited muscle disorders of childhood (1). This progressive, X-linked, recessive, muscle-wasting disorder is caused by mutations in the *DMD* gene and is one of the most severe types of muscular dystrophy with childhood onset (2–5). Individuals with DMD experience progressive muscle weakness caused by the absence or severe reduction of dystrophin, a muscle protein crucial to maintain strength, stability, and function of myofibers (6). The global prevalence of DMD has been estimated as 7.1 cases/100,000 males and 2.8 cases/100,000 in the general population, with a pooled global birth prevalence of 19.8 cases/100,000 live male births (7). Similarly, the birth prevalence has been reported as 15.9 cases per 100,000 live male births in the USA and 19.5 cases per 100,000 live male births in the UK (2). Skeletal muscle damage and degeneration in DMD start early in life and result in an elevation in serum creatine kinase (CK), progressive muscular weakness, motor delay, loss of ambulation, respiratory impairment, and cardiac complications (2, 4, 8). A proportion of DMD patients also present with neurocognitive dysfunction, language delay, autistic spectrum disorder, attention deficit-hyperactivity disorder, and other symptoms outside of skeletal muscle. The phenotypic severity in skeletal and cardiac muscle and the expression of neurocognitive symptoms in DMD can vary based on the underlying mutation type. The complications of the disease lead to decreased survival, with death usually occurring due to cardiac or respiratory failure (2). Early implementation of a multidisciplinary standard of care has resulted in improved survival, and the median life expectancy for patients with DMD born after 1990 is now estimated to be 28.1 years, compared with 18.1 years for patients born before 1970 (9).

The emergence of new therapeutic approaches, including dystrophin restoration strategies (2, 4–6, 10, 11) has highlighted the importance of an early and genetically-confirmed diagnosis, with a growing emphasis on detecting the very early signs of impairment in young boys with DMD (12–14). However, there is a persistent diagnostic delay, and the diagnosis of DMD is, on average, not made until 4–5 years of age, a delay of up to two years from the appearance of the first symptoms to a confirmed diagnosis (15–17). This delay has not significantly improved in the past two decades, despite the initiatives of public health and patient organizations to raise professional awareness (12). There are many compelling reasons to shorten the time to a definite diagnosis in DMD, including timely genetic counseling and assessment of carrier status, initiation of multidisciplinary standard care, timely initiation of corticosteroids, precise genetic mutation characterization to allow access to newly approved drugs targeted at specific mutations, an opportunity to participate in clinical trials, avoid unnecessary and expensive or invasive tests, and improve the family psychological burden of a “diagnostic odyssey”.

In this comprehensive review of the literature, we describe the very early signs of impairment in DMD by the age of onset and type and discuss the importance of their recognition to avoid diagnostic delay and improve early diagnosis of the disease.

## 2. Methods

We searched PubMed for records of articles in English published from January 1, 2010, to November 30, 2022, reporting evidence of the effects of early diagnosis or diagnostic delay on developmental milestones in patients with Duchenne muscular dystrophy. The following search terms (limited to title/abstract) were used: Duchenne muscular dystrophy, DMD, or Duchenne AND any of the following search terms (limited to title/abstract): development/ developmental milestones/, early clinical signs, early detection or diagnosis or signs/ diagnostic delay/ time to diagnosis/ diagnostic timing.

## 3. Results

**Figure 1:**
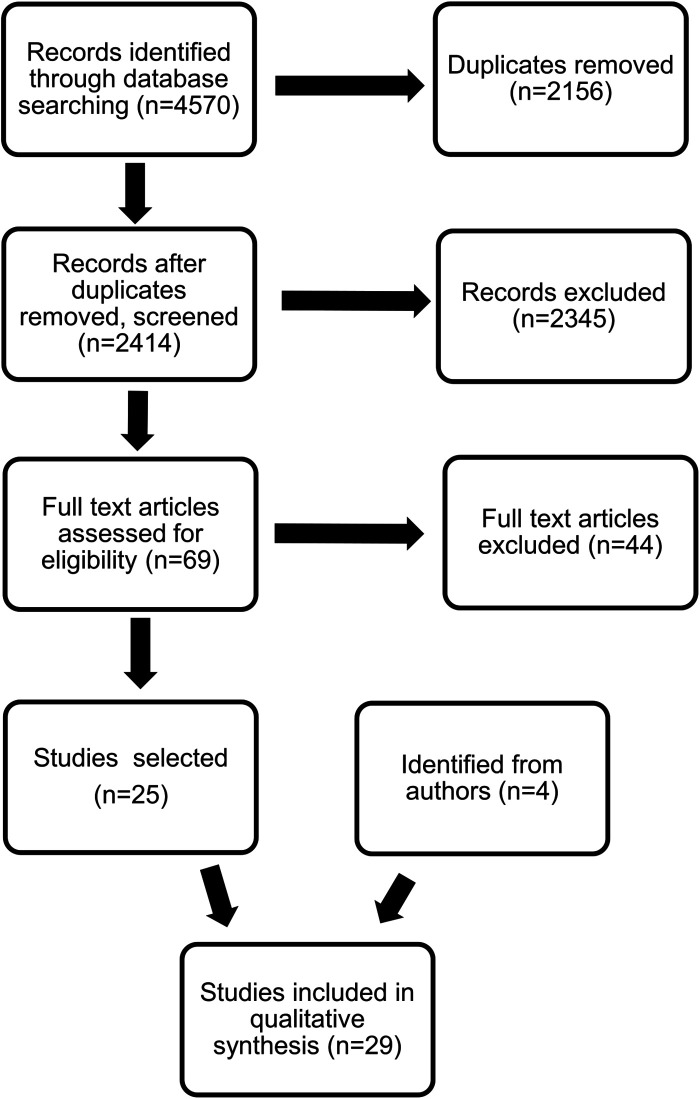
PRISMA diagram of the selection process on the publications included in the qualitative analysis.

The search identified 2,414 records, with the great majority focusing on the diagnostic pathway without any clear description of the clinical signs leading to diagnosis or early developmental scales. A total of 2,123 were single case reports or otherwise non-relevant, 12 articles were not in English, and 210 were not studies in humans. Sixty-nine abstracts were selected for further consideration, and the full papers were reviewed, leading to the identification of an additional 11 studies published before 2010 and not detected by the original search. Twenty-nine papers related to the issue of diagnostic delay in DMD, 25 from the original search and another 4 from secondary identification by the authors, were included in the final selection and will be reported in this review (Figure 1, Table 1).

**Table 1:** Summary of included publications reporting early diagnosis, early neurological signs and/or developmental milestones.

Author, year	Study design	Age range considered (years)	Age at diagnosis (mean, years)	Cognitive assessment	Assessment of milestones	Neurological assessment	*N*
Aartsma-Rus et al. (12)	EBM consensus and systematic review	NA	NA	no	no	no	NA
Allen et al. (18)	Case series	2–16	NA	no	no	yes	6
Annexstad et al. (19)	Research article (retrospective)	0–18	3.9	no	yes	yes	73
Chieffo et al. (20)	Research article (prospective)	2–6	NA	yes	yes	no	41
Chieffo et al. (21)	Research article (prospective)	4–6	NA	yes	limited to language	no	40
Ciafaloni et al. (22)	Research article (retrospective)	2.5	4.9	yes	no	yes	156
Connolly et al. (23)	Research article (prospective)	0–3	NA	yes	yes	yes	24
Connolly et al. (24)	Research article (prospective)	1–2	NA	yes	yes	no	19
Coratti et al. (25)	Research article (prospective)	<6	NA	no	no	yes	153
Counterman et al. (26)	Research article (retrospective)	1–4	4.43	no	no	no	1282
Crossnohere et al. (27)	Research article (retrospective)	2	4	no	no	no	65
Cyrulnik et al. (28)	Research article (prospective)	4–14	NA	yes	limited to language	no	130
D'Amico et al. (13)	Research article (retrospective)	2–5	3.4	no	no	yes	384
Darmahkasih et al. (29)	Research article (retrospective)	NA	NA	yes	no	no	698
Gissy et al. (30)	Research article (retrospective)	NA	NA	no	yes	yes	1110
Houwen van Opstal et al. (31)	Research article (retrospective)	NA	NA	no	no	yes	232
Lee et al. (32)	Research article (retrospective)	ND	4.9–6.6	yes	yes	no	40
Ma et al. (33)	Research article (retrospective)	3.9	6.8	no	yes	yes	152
Mirski and Crawford, (16)	Research article (retrospective)	NA	5.1	yes	yes	no	179
Norcia et al. (14)	Research article (retrospective)	0–2	no	no	yes	no	134
Pane et al. (34)	Research article (prospective)	0.5–3.5	no	yes	yes	yes	81
Pereira et al. (35)	Research article (retrospective)	2–12	no	no	no	yes	128
Sarrazin et al. (36)	Research article (retrospective)	NA	NA	no	yes	no	263
Soim et al. (37)	Research article (retrospective)	NA	4.1–4.9	no	no	yes	325
Takeuchi et al. (38)	Research article (retrospective)	NA	NA	yes	yes	yes	46
Schiava et al. (39)	Research article (prospective)	4–8	4.5	no	yes	no	196
Thomas et al. (17)	Research article (retrospective)	2–5	4.9	no	no	yes	221
Van Dommelen et al. (40)	Research article (prospective)	NA	NA	no	yes	no	74
van Ruiten et al. (41)	Research article (retrospective)	NA	4.3	no	no	no	10

EBM, evidence-based medicine; NA, not available; N, number of patients assessed.

## 4. Developmental milestones and early gross motor function in DMD

Motor symptoms such as difficulties in running, jumping, rising from the floor (Gower’s sign), struggling to hop or climb stairs, frequent falls or trips, abnormal and/or waddling gait or toe walking, together with calf hypertrophy or muscle pain or cramps, are the most typical signs classically identified in DMD children around the age of 3–4 years and prompting further investigations leading to the diagnosis of DMD (2, 4, 7, 12, 14, 40). Over the last few years, there has been increasing evidence that children with DMD often experience delays in early developmental milestones compared with typically-developing children (2, 16, 28, 42, 43). (Table 2). Between 36% and 67% of children with DMD were reported to be late in achieving developmental milestones less commonly recognized as typical of DMD, such as speaking, forming sentences, bladder or bowel training, or reading (28).

**Table 2:** Typical early signs and symptoms of Duchenne muscular dystrophy.

Motor	
Delayed walking	Toe walking
Difficulty rising from the floor	Frequent trips, falls, or clumsiness
Gower’s sign on rising from the floor	Muscle pain or calf hypertrophy or cramps
Difficulty climbing or descending stairs	Gross motor delay
Difficulty running, walking or climbing	Hypotonia
Inability to jump	Loss of motor skills
Abnormal gait	Inability to keep up with peers
Waddling gait	Poor head control
Nonmotor	
Neurocognitive deficits	Learning disabilities
Autism spectrum disorder	Speech delay / articulation issues
Failure to thrive or poor weight gain	Behavioral issues
Other	
Male sex	Elevated creatine kinase levels
Family history	Elevated aminotransferases or lactate dehydrogenase

The delay includes the first milestones achieved after birth, such as poor head control in infants, sitting without support, crawling on hands and knees, standing alone, walking with assistance, and walking independently by 18 months. A recent study using questionnaires completed by the parents shows that it is possible to differentiate between young males with DMD and controls as early as a few months after birth (40). The presence of developmental delay was already evident at 2–3 months; a higher proportion of males with DMD failed to attain milestones of gross/fine motor activity, adaptive behavior, personal/social behavior, and communication. The differences in attaining developmental milestones relating to gross motor activity increased with age. Other studies also reported similar findings in early neurodevelopmental milestones by interviewing families or extracting information from clinical notes (14). In the DMD subjects, walking independently was achieved at a mean age of 16.35 months, compared with 12.26 months in the control group. Meanwhile, 16.9% of boys with DMD did not achieve independent walking by 18 months, whereas all controls achieved that milestone (*p* < 0.001) (14).

Other studies using structured neurodevelopmental assessments have also confirmed the early differences in neurodevelopment (23, 24, 34). The Bayley-III scales were prospectively used to assess motor and cognitive development in boys with DMD aged ≥1 month but <3 years old (mean 1.9 years) (23). Mean motor composite, gross motor-, and fine motor function-scaled scores were lower than in typically-developing children, as were mean cognitive comprehensive, receptive language, and expressive language assessments. Gross motor scores further declined over 6 and 12 months of follow-up (24). However, cognitive and language scores did not change significantly at 6 or 12 months, and fine motor scores improved over one year.

Comparable findings were also reported before the age of 4 years in young DMD boys (age 7–47 months) in a study that used the Griffiths Scale of Mental Development (34). The mean Development Quotient (DQ) in DMD was 87, approximately one standard deviation below the mean, and almost a third (32.1%) of DMD boys had a borderline DQ (between 84 and 70), and 12.3% had an abnormal DQ <70) (34).

Developmental delays may be associated with a number of factors, including specific genotype, race/ethnicity, and sociodemographics (13, 14, 22, 26). Moreover, a rapidly increasing number of studies suggest an important role of specific brain dystrophin isoforms in influencing early aspects of gross motor and neurocognitive development (14). Patients with mutations upstream of exon 44 have reduced expression of Dp 427 only, mutations after exon 51 affect both Dp427 and Dp140, and mutations after exon 63 have reduced expression of Dp427, Dp140, and Dp71. Mutations between 44 and 51 have recently been reported separately, as the involvement of Dp 140 in these patients is not always clear. Norcia et al. retrospectively assessed the age when early motor milestones were achieved in DMD boys and found an increasingly high risk of delay in achieving independent sitting and walking in patients with mutations predictive of the involvement of different brain dystrophin isoforms (14). Sitting independently was achieved at a mean of 7.04 months in boys with DMD and in 7.07 months by the typically-developing control group. However, 9.7% of boys with DMD did not achieve independent sitting by 9.4 months, which ranged from 2.2% in the boys with mutations before exon 44%–33.3% in those beyond exon 63. In the overall DMD cohort, values ranged between 9.5% in boys with mutations between exon 44 and 51 and 42.9% in boys with mutations beyond exon 63.

Similar findings were observed in the study using the Griffiths developmental scales (34). Boys with mutations upstream or in exon 44 had a higher DQ than those with mutations downstream exon 44, which are known to be associated with additional involvement of dystrophin isoforms expressed at high levels in the brain. This further suggests that the site of mutation and the involvement of dystrophin isoforms may modulate aspects of global development.

While the cumulative effect of loss of isoforms is more evident in language and the more ‘cognitive’ aspects of the neurodevelopmental scales, this is also seen in gross motor aspects, as also confirmed by recent studies in preschool children showing that *DMD* mutations expected to impact on dystrophin isoform production were differentially affecting motor function in DMD. When DMD boys were subdivided considering the expected effects of *DMD* mutation on dystrophin isoform expression, there was a reduced mean peak North Star ambulatory assessment (NSAA) score in those lacking Dp140 and Dp71, with a clear cumulative effect of loss of isoforms (44, 45).

It is of note that in these children, some motor milestones and some aspects of motor function, such as the ability to go up and down stairs, were achieved at a later age compared to their peers, suggesting that delayed achievement of milestones and functional aspects is likely to be due at least partly to the involvement of brain dystrophin isoforms (16). In contrast, motor skills related to muscle weakness, such as running or hopping, were generally not achieved in DMD boys, irrespective of age, unless they were treated with steroids or other therapeutic agents.

## 6. Other genotype-phenotype associations

The advent of clinical trials and commercially available therapies targeting specific groups of mutations, such as nonsense mutations or groups of deletions amenable to skipping individual exons, have highlighted possible differences among these subgroups. Several recent studies exploring functional changes in patients with different subgroups of mutations amenable to skip individual exons (mainly skipping exon 44, 45, 51 or 53) have reported that, while the individual subgroups may not show significant differences compared to the mean values of the whole DMD cohort, there are significant differences among the individual subgroups that become more obvious with increasing follow up (25, 46, 47). For example, two recent studies using the NSAA and the six-minute walk test (6MWT) over 3 years showed that boys with mutations amenable to skip exons 51 and 53 have a faster functional decline and increased risk of losing ambulation over a 36-month period than other subgroups, while those amenable to skip exon 44 have higher scores at baseline and less rapid decline (46).

Several other studies have reported similar findings, with deletions skippable exon 51 or 53 being associated with an overall more severe phenotype, more rapid progression, and earlier age at loss of ambulation (47), as well as cardiac and respiratory impairment (47, 48).

So far, there are no systematic studies reporting differences in early motor milestones in these subgroups of deletions.

## 7. Diagnosis and diagnostic delay

The mean age at diagnosis in DMD is still between 4 and 5 years, representing a delay of up to two years from the first appearance of symptoms (13, 15–17, 22, 40). Ciafaloni et al. reported that the first signs of symptoms of DMD in a cohort of boys without a known family history were observed at a mean age of 2.5 years, leading to evaluation by a primary care provider at a mean of 3.6 years (22). However, initial CK testing was not undertaken until a mean of 4.7 years, followed by a definitive diagnosis at a mean of 4.9 years. That is, there was a delay of approximately 2.5 years from the onset of symptoms to a definitive diagnosis of DMD.

As part of the FOR-DMD study, Investigators of the Muscle Study Group found that the mean age at ﬁrst parental concern was 29.8 months, with motor development delays, walking difficulties, and speech delays the most common presenting symptoms. There was a mean diagnostic delay of 25.9 months before a genetic diagnosis at 53.9 months (39). Of note, the mean diagnostic delay after an incidental ﬁnding of elevated CK level was 6.4 months.

Studies suggest that the time to diagnosis is much shorter following an incidental/accidental finding of elevated CK levels (13, 34, 39). D'Amico et al. investigated the age at diagnosis of DMD in Italy in 384 boys diagnosed with DMD from 2005 to 2014 (13). The mean age at ﬁrst medical contact at which the suspicion of DMD was raised was 31 months, with confirmation of the diagnosis at a mean age of 41 months. The overall mean age at diagnosis in Italy was approximately 10–12 months lower than that reported in other countries. This is partly due to the fact that the most frequent finding leading to a suspicion of DMD was an incidental ﬁnding of consistently elevated serum CK level detected on routine blood testing, which is often performed for CK and transaminases in Italy in children with vomiting, diarrhea, prolonged fever, or before general anesthesia. In cases where elevated CK level was the initial finding, a diagnosis was achieved earlier (mean 25 months) than in children presenting with a developmental delay (mean 30 months). Of note, DMD was not suspected until a mean of 45–49 months in children showing toe walking or muscle weakness (13).

There are multiple reasons for diagnostic delay, which are outlined below.

### 7.1. Delay in identifying early clinical signs

Despite the increasing evidence of the importance of early clinical signs in DMD, the diagnostic process is often still not initiated until the more typical signs and symptoms, such as difficulty running/walking, difficulty climbing/descending stairs, toe walking, hypertrophic calves, and the presence of Gower’s sign, become obvious (2, 4, 12). As well, in many cases, the time between the detection of these signs and the final diagnosis is still very long, with pediatricians opting for a wait-and-see approach or referral to orthopedics or other non-neuromuscular specialists.

### 7.2. Delay in ordering serum CK testing

In cases where the physician initiates a diagnostic workup for global developmental delay, CK should be routinely included, as it is an inexpensive and universally available test that is very useful in directing the diagnosis toward a primary neuromuscular cause. Yet, CK testing is frequently not included in the early diagnostic workup of children with global developmental delay or when motor symptoms are accompanied by more neurocognitive deficits, typically not associated with a primary neuromuscular disorder or recognized as typical of a primary neuromuscular disease.

### 7.3. Wrong interpretation of elevated ALT/AST

Creatine kinase testing is a sensitive screening tool in the diagnostic pathway for DMD (2, 10, 12, 16, 49). and should always be performed whenever elevated alanine aminotransferase (ALT) and aspartate aminotransferase (AST) are found, especially if gamma-glutamyl transferase (GGT) has not been tested or is normal. Elevated CK should always prompt the need for a detailed neurological assessment and further investigation for DMD. However, there are still reported cases in which elevated AST and ALT levels were thought to be related to liver involvement and led to a referral to a hepatologist and, in some cases, to liver biopsy, leading to a delay in the diagnosis of DMD. The use of GGT in distinguishing a primary liver rather than a muscle disorder should be emphasized.

### 7.4. Delay in genetic confirmation

In the past, genetic confirmation of DMD, especially for the forms due to small mutations, was not always readily available. However, newer generations of testing, including next-generation sequencing (NGS), have become available and more accessible, allowing a diagnosis to be reached without further delay. As approximately 70% of individuals with DMD have a single-exon or multi-exon deletion or duplication in the *DMD* (dystrophin) gene, testing for dystrophin gene deletion and duplication by multiplex ligation-dependent probe amplification (MLPA) is still in many countries a frequent first test to confirm DMD (2, 4, 10, 12). There are also many programs provided by organizations, such as the Parent Project Muscular Dystrophy (PPMD), Decode Duchenne, and the Detect initiative, that now offer free DNA testing for DMD.

## 8. Reducing the time to diagnosis

**Figure 2:**
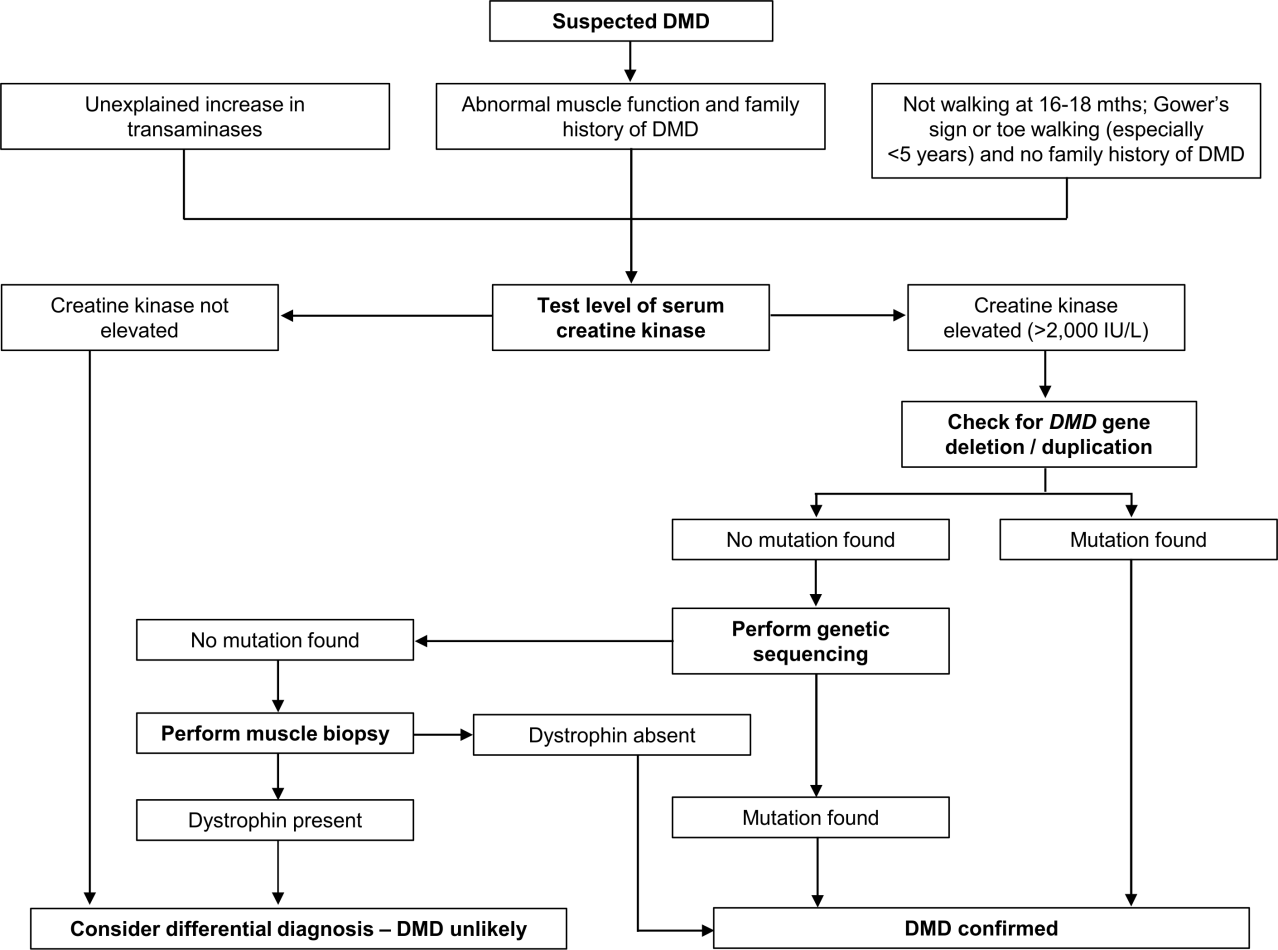
Diagnosis of Duchenne muscular dystrophy (DMD). Algorithm based on data from Birnkrant et al. (2), D'Amico et al. (13), and Ciafaloni et al. (22).

Establishing a timely and accurate diagnosis is a crucial aspect of the effective care of DMD (2). (Figure 2). Performing timely management and interventions before irreversible muscle damage occurs has become a focus of renewed interest as new therapies become available that make reducing the diagnostic delay and planning appropriate intervention even more important (6). Although early cognitive and motor development milestones are common presentations of DMD, they are currently not used to their maximal potential in identifying children with a diagnosis suspicious of DMD (16, 40). Failure to recognize early signs and symptoms of early DMD, which may be nonspecific, is still often a cause of delayed diagnosis (22).

Early identification of infants at risk for developing DMD by performing routine newborn screening (NBS) for DMD has been piloted in several programs (15, 50–53). and has recently gained momentum, as demonstrated by the submission in June 2022 of a nomination package to add NBS for DMD to the Recommended Uniform Screening Panel (https://www.hrsa.gov/advisory-committees/heritable-disorders/rusp). Nevertheless, NBS remains controversial (2, 4, 15). As NBS is generally recommended for genetic disorders with neonatal-onset, for which there is robust evidence of the benefits of early treatment on outcome. These criteria are not completely met for DMD.

While the difficulties of having DMD as part of routine NBS continue to be investigated, alternative screening options have been proposed to reduce the delays in the clinical diagnosis and specialist referral and to improve early genetic confirmation. Proposed approaches include increasing awareness among health providers and reducing long waiting times to see a specialist (12). However, the overall effort to improve awareness among pediatricians, pediatric neurologists, and other healthcare professionals is unlikely to reach all professionals involved unless part of a structured program.

The National Task Force for Early Identification of Childhood Neuromuscular Disorders (https://childmuscleweakness.org/), in their guides for primary care providers and other early intervention specialists, reinforces the difference that early diagnosis makes. The Task Force sets out steps to identify pediatric muscle weakness and signs of neuromuscular disease, with guidance for primary care providers to take the opportunity for developmental surveillance at every health supervision visit and at 9-, 18-, and 24- or 30-month visits (43). They recommend that providers Listen, Observe, Evaluate, Test, and Refer to ensure speedy diagnosis and enable early access to treatment.

As testing CK levels provides a simple, inexpensive, and accurate guide to support a suspicion of DMD, assessing CK levels in primary rather than secondary care would significantly reduce diagnostic delay. This could be achieved by performing CK testing in the presence of neurodevelopmental delay (walking delay >18 months, delayed speech, or global developmental delay) (2, 4, 12, 13, 16). Once a suspicion of DMD has been identified, the time to reach a definitive diagnosis of DMD is relatively short (12).

This approach would definitely help to identify a larger number of DMD boys but has the disadvantage that delayed speech or walking are very common among typically-developing boys and would be a burden for primary care health professionals, generating a high number of negative results, i.e., boys with speech or walking delays and normal CK levels.

Increasing awareness among primary care providers on the need to focus on more specific early signs of muscle weakness that are more suggestive of DMD (difﬁculties in getting up from the ﬂoor, toe walking, calf hypertrophy), even in children not manifesting signs of global development delay, should increase the sensitivity to detect DMD or, more generally, a neuromuscular disorder, and reduce the number of negative results (13).

A parallel approach should be to increase awareness of the association between increased CK levels and neuromuscular disorders when GGT is normal, to avoid wrong referral and shorten the diagnostic process once elevated levels of CK are picked up incidentally by the primary care provider (2, 4).

## 9. Conclusions

In recent years, new disease-modifying treatments for DMD have become available, are in development, or nearing approval. The consensus is that most treatments have the best chance of producing a greater beneficial effect when initiated early in the course of the disease and before significant muscle degeneration and fibrosis have occurred. Diagnosis of DMD continues to be delayed on average until age 4–5, with no significant change in this diagnostic delay in the past two decades despite significantly improved access and precision in genetic testing. Failure to recognize the early signs of DMD is a leading cause of this diagnostic delay.

Here we have provided a comprehensive review of the early signs in DMD and highlighted new evidence showing how neurocognitive and developmental symptoms are more common than previously thought, occurring very early in infants and children with DMD. Until universal NBS becomes appropriate and available for DMD, the best mitigation strategy for improving the age at diagnosis is to increase awareness of all of the early symptoms of DMD, including the “less classic” ones presenting very early, and to encourage early CK screening in all boys who present signs of developmental delay and specific motor test abnormality at their routine pediatrician visits.
